# Mucosal Vaccination Against Periodontal Disease: Current Status and Opportunities

**DOI:** 10.3389/fimmu.2021.768397

**Published:** 2021-12-02

**Authors:** Victor Vaernewyck, Boaz Arzi, Niek N. Sanders, Eric Cox, Bert Devriendt

**Affiliations:** ^1^ Laboratory of Immunology, Department of Translational Physiology, Infectiology and Public Health, Faculty of Veterinary Medicine, Ghent University, Merelbeke, Belgium; ^2^ Department of Surgical and Radiological Sciences, School of Veterinary Medicine, University of California, Davis, CA, United States; ^3^ Veterinary Institute for Regenerative Cures (VIRC) School of Veterinary Medicine, University of California, Davis, CA, United States; ^4^ Laboratory of Gene Therapy, Department of Veterinary and Biosciences, Faculty of Veterinary Medicine, Ghent University, Merelbeke, Belgium

**Keywords:** periodontal disease, gingivitis, periodontitis, mucosal vaccine, periodontal vaccine

## Abstract

Approximately 9 out of 10 adults have some form of periodontal disease, an infection-induced inflammatory disease of the tooth-supporting tissues. The initial form, gingivitis, often remains asymptomatic, but this can evolve into periodontitis, which is typically associated with halitosis, oral pain or discomfort, and tooth loss. Furthermore, periodontitis may contribute to systemic disorders like cardiovascular disease and type 2 diabetes mellitus. Control options remain nonspecific, time-consuming, and costly; largely relying on the removal of dental plaque and calculus by mechanical debridement. However, while dental plaque bacteria trigger periodontal disease, it is the host-specific inflammatory response that acts as main driver of tissue destruction and disease progression. Therefore, periodontal disease control should aim to alter the host’s inflammatory response as well as to reduce the bacterial triggers. Vaccines may provide a potent adjunct to mechanical debridement for periodontal disease prevention and treatment. However, the immunopathogenic complexity and polymicrobial aspect of PD appear to complicate the development of periodontal vaccines. Moreover, a successful periodontal vaccine should induce protective immunity in the oral cavity, which proves difficult with traditional vaccination methods. Recent advances in mucosal vaccination may bridge the gap in periodontal vaccine development. In this review, we offer a comprehensive overview of mucosal vaccination strategies to induce protective immunity in the oral cavity for periodontal disease control. Furthermore, we highlight the need for additional research with appropriate and clinically relevant animal models. Finally, we discuss several opportunities in periodontal vaccine development such as multivalency, vaccine formulations, and delivery systems.

## Introduction

Periodontal disease (PD) is an infection-induced chronic inflammatory disease that affects the tooth-supporting tissues, which are collectively known as the periodontium. In gingivitis, the initial reversible form of PD, inflammation is confined to the gingival epithelium and the connective tissue. If not treated properly, this inflammation can spread to the deeper components of the periodontium, including the alveolar bone, leading to periodontitis, the irreversible form of PD (1). Periodontitis can be further classified into four stages (I, II, III, or IV) and three grades (A, B, or C). Staging is based on severity, complexity, extent, and distribution; while grading is based on the rate of progression, anticipated treatment response, and effects on systemic health (2).

Based on the World Health Organization’s oral health database, about 90% of adults have some form of PD (gingivitis or periodontitis) (3). While this estimate is interesting, it does not reflect the clinical impact of PD since many of these cases are asymptomatic. However, the clinical importance of PD is corroborated by the high global prevalence of severe periodontitis, which was estimated at 9.8% by the Global Burden of Disease (GBD) study (4). PD is also highly prevalent in adult dogs, cats, minipigs, and non-human primates, with anesthetized examination studies reporting prevalences of 86.5% to 100% (5–12).

PD typically leads to halitosis, oral pain or discomfort, and periodontal damage which can result in tooth loss (1). Moreover, periodontitis may have a substantial effect on systemic health. Epidemiological, clinical interventional, and experimental studies have provided compelling evidence that periodontitis adversely impacts systemic health in humans. However, clear confirmation that successful treatment of PD can reduce the risk or incidence of PD-associated conditions like atherosclerosis and type 2 diabetes mellitus is lacking (13, 14). Veterinary research into the extra-oral effects of periodontitis remains limited, but a growing body of literature suggests similar deleterious effects on systemic health in animals with periodontitis (15–21). In addition to the oral and systemic disease burden, PD also imposes a significant economic burden. The global annual cost (direct and indirect) of human dental diseases was estimated at 544 billion USD in 2015, which is largely attributed to PD and caries (22).

Gingivitis is clinically characterized by gingival redness, swelling, and susceptibility to bleeding. Periodontitis implies loss of gingival attachment to the tooth, degradation of the periodontal ligament and loss of alveolar bone (1). This destructive process is associated with the presence of subgingival polymicrobial communities and a dense immuno-inflammatory infiltrate in the periodontium, which can be explained by the polymicrobial synergy and dysbiosis model. This model describes PD as a continuous cyclic process where dysbiotic polymicrobial communities within the subgingival dental plaque induce an immune response that is ineffective, uncontrolled, and destructive in a susceptible host. The resulting inflammatory environment and tissue degradation exacerbate dysbiosis by selectively providing nutrients to inflammophilic bacteria, thereby generating a self-sustained feed-forward loop that perpetuates the disease (23).

The PD-associated polymicrobial communities are nososymbiotic rather than pathogenic, as their collective pathogenic potential depends on both the outcome of interbacterial interactions and host susceptibility. Consequently, the simple dichotomous characterization of microbes as either commensals or pathogens is not adequate to represent the continuum from commensalism to pathogenicity. Instead, several functional categories have been established such as keystone pathogens, accessory pathogens, pathobionts, and homeostatic commensals (24) Keystone pathogens such as *Porphyromonas gingivalis* have a disproportionately large influence on the quantitative and qualitative microbial composition, thus acting as a keystone of their community’s structure. These changes may be induced directly *via* interspecies interactions and indirectly through subversion of the host immune response (25–29). In addition, there are accessory pathogens (e.g., *Streptococcus gordonii*), which are generally perceived as symbiotic commensals, but they can promote the virulence of disease-associated organisms by supporting the nutrition and colonization of keystone pathogens (30–33). Next, there are pathobionts (e.g., *Treponema denticola, Tannerella forsythia*, and *Fusobacterium nucleatum*), which are inflammophillic commensals that can become pathogenic when host-microbe homeostasis is disrupted under certain conditions, such as inflammation, antibiotic treatment, tissue damage, dietary shifts, and immune deficiencies (34). The fourth major group are homeostatic commensals (e.g., *Streptococcus cristatus*), which are commensals that stabilize eubiotic communities by directly antagonizing potentially pathogenic microbes or by inducing antimicrobial peptides that preferentially target potential pathogens (35–38).

Current PD control measures heavily rely on the removal of dental plaque and calculus by mechanical debridement. This is usually limited to toothbrushing, interdental cleaning, and non-surgical periodontal therapy (scaling and root planing), although open flap debridement is occasionally needed (39). While these procedures can prevent the formation of a disease-triggering dysbiotic biofilm, it does not directly affect the latent dysregulated inflammatory cascade in susceptible hosts. Therefore, mechanical debridement requires constant repetition and provides variable prognoses depending on patient compliance and susceptibility (40). Consequently, several adjuncts to mechanical debridement have been proposed to enhance treatment outcomes. This includes pocket reduction surgery (41), regenerative surgery (42), laser therapy (43), and local antimicrobials such as doxycycline or chlorhexidine (44, 45). However, current adjunct therapy mostly relies on systemic antibiotics; which typically consists of a broad-spectrum antibiotic alone or in combination with an antibiotic that targets Gram-negative bacteria (39).

The rationale for administration of systemic antimicrobials as an adjunct to non-surgical therapy is to reduce the number of pathogenic bacteria in deep pockets, surface irregularities, furcation areas, and those that have entered the bloodstream. However, the use of antimicrobials is only justified in specific cases of periodontitis, since biofilm-associated infections are difficult to treat with antibiotics, and the use of antimicrobial agents promotes the development antimicrobial resistance (46). Despite these considerations and established clinical guidelines, several studies indicate that systemic antibiotics are still regularly used to control PD without appropriate indications (47–51). This injudicious use of antibiotics is alarming, especially considering the ubiquity of PD and the extra-oral distribution of systemic antibiotics, as this contributes to the development of antimicrobial resistance (46). Antimicrobial resistance has evolved as one of the most urgent threats to public health, causing treatment failures, prolonged hospital admissions, and increases in healthcare costs (52). Moreover, several studies indicate high and increasing levels of antimicrobial resistance in subgingival PD-associated bacteria, further exposing the unsustainability of antibiotics-based PD management (53–63). Another drawback of antimicrobials is their non-specific effect on both pathogenic (e.g., keystone and accessory pathogens) and protective oral bacteria (homeostatic commensals) (46).

Since the host inflammatory response acts as main driver of tissue destruction and simultaneously exacerbates dysbiosis, it can be reasoned that adjuncts to mechanical debridement should not rely on nonspecific bacterial clearance by systemic antibiotics, but rather on the alteration of host immune responses. Traditional anti-inflammatory drugs, such as corticosteroids and non-steroidal anti-inflammatory drugs do not offer significant long-term benefits and are precluded for prolonged periodontal treatment due to their adverse effects (64–67). However, several promising alternatives have been proposed, including specialized pro-resolving mediators, complement inhibitors, and anti-cytokine therapies. Pro-resolving mediators are physiological agents such as resolvins, lipoxins, and protectins which accelerate the resolution of inflammation (68). The topical application of such mediators can protect against bone loss in rabbits, rats, and miniature pigs following experimental induction of periodontitis (68–73). Complement inhibitors, on the other hand, aim to suppress the complement system which is overactivated in periodontitis (74–77). Recent studies indicate that topical or systemic administration of Cp40, an inhibitor of the complement component C3, inhibits naturally occurring periodontitis in non-human primates (76, 77). A third novel approach to immune response modulation are anti-cytokine therapies, which involve the use of neutralizing monoclonal antibodies or receptor antagonists to block the action of proinflammatory cytokines that play a role in periodontitis (78–84). Studies in non-human primates with experimentally induced periodontitis found that local injections with antagonists of interleukin 1 and/or tumor necrosis factor protected against PD-associated tissue loss (78–80, 83).

The three aforementioned strategies could be relevant for future treatment of periodontitis or even its short-term prevention in high-risk individuals; however, these methods seem less useful for long-term prophylaxis (23). In contrast, periodontal vaccines may contribute to long-term prophylaxis, by preventing the subversion of the immune system by keystone PD pathogens, avoiding and reverting dysbiosis, and averting destructive hyperinflammation (23). In addition, periodontal vaccines might discourage the use of antibiotics (46). Efficacious periodontal vaccines will need to elicit protective antibody responses in the oral cavity that are specific for PD-inducing bacteria. Local antibody responses in the oral cavity rely on both systemic (IgG) and mucosal immunity (secretory IgA, SIgA). IgG within the oral cavity mainly originates from the blood circulation by passive leakage *via* the gingival crevicular epithelium, while the SIgA is locally produced in the salivary glands by activated B cells that migrated from the mucosa-associated lymphoid tissues (MALT) (85). Hence, effective periodontal vaccines must induce both systemic and mucosal immunity in the oral cavity, which has proved difficult with traditional vaccination strategies. This is evident by the current lack of a human periodontal vaccine, while the first vaccines against PD, including the Inava Endocorps vaccine, were already developed in the early twentieth century (86). Similarly, there have been no veterinary periodontal vaccines available since the production of the Porphyromonas-denticanis-gulae-salivosa vaccine against PD in dogs was halted in 2011 due to its unsatisfactory long-term effects on the disease (87). Fortunately, there has been major progress in the design of mucosal vaccines, offering new methods to induce protective immunity in the oral cavity (88). Another area of improvement is the antigen selection, which is aided by the growing understanding of the polymicrobial compositions and interactions. Based on the current knowledge, successful periodontal vaccines may require multiple specific antigenic targets from different PD-associated bacteria (23). In this review, we provide a comprehensive overview of the current status and future directions of mucosal vaccination against PD.

## Mucosal Vaccination Against Periodontal Disease

**Figure 1:**
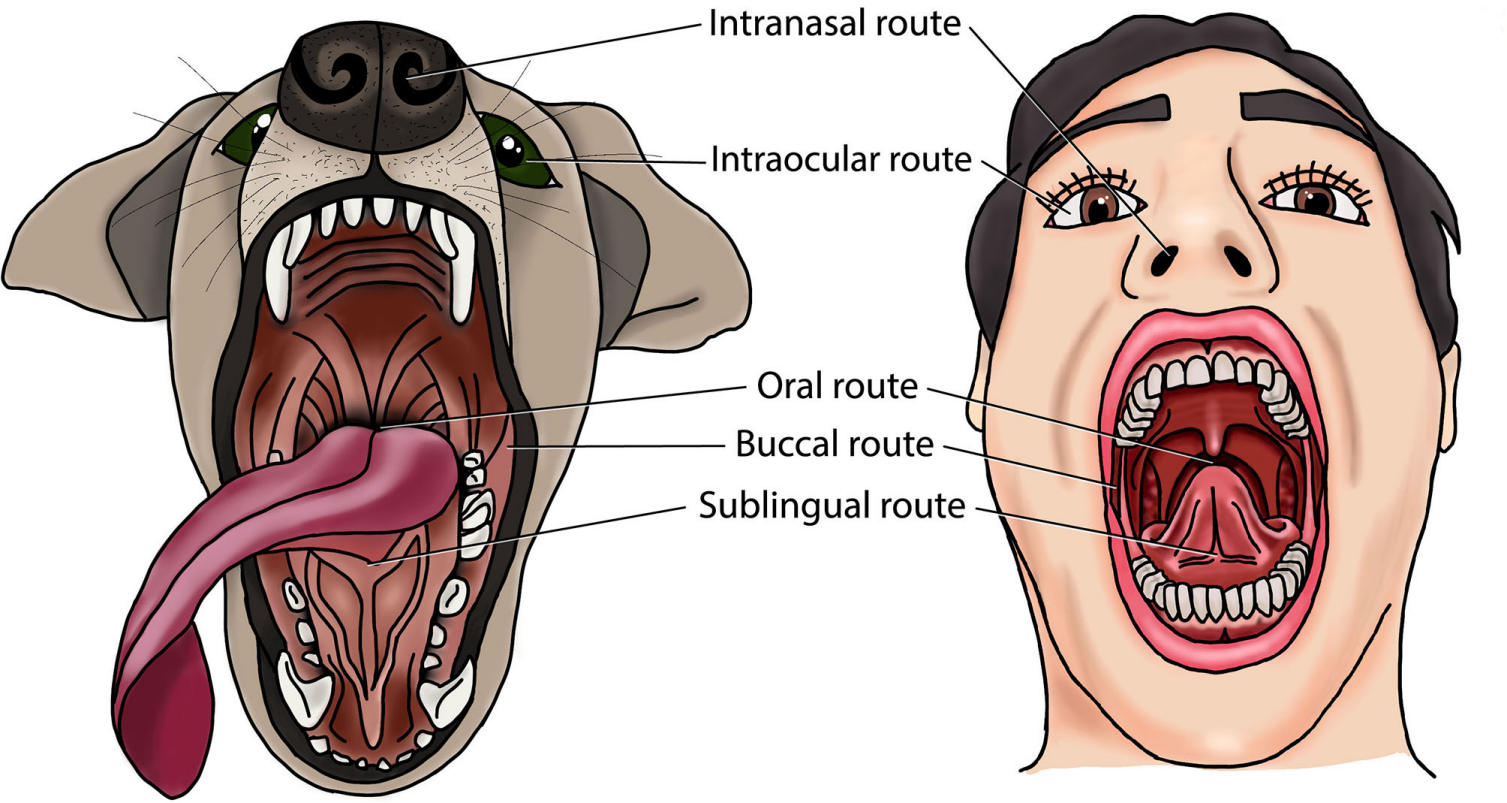
Mucosal routes for periodontal disease vaccination in dogs and humans.

Mucosal vaccines are more likely to protect against PD than systemic vaccines, since they are generally more successful in simultaneously inducing IgG and salivary SIgA in the oral cavity (88). Indeed, all eleven preclinical studies that evaluated this reported more dual immunity in the oral cavity after mucosal vaccination compared to systemic vaccination (89–99). Furthermore, several studies reported protection against experimental PD-associated bone loss or gingival swelling/abscessation by mucosal immunization (91, 100–115). While these data support the rationale for mucosal PD vaccines, it must be noted that all but two of these studies used rodent PD models (113, 116). Rodents have been popular because of their low cost, manageability, prompt availability, and ease of housing. However, rodents have major limitations as translational model of human PD. First, there are marked differences in periodontal anatomy and oral microbiota between rodents and humans. Second, PD does not occur spontaneously in rodents, requiring experimental PD induction with allochthonous PD bacteria such as *P. gingivalis* (117). Third, clinical parameters such as bone loss are difficult to measure and interpret in small animals like mice, which is further complicated by the lack of standardization (118). Finally, the lymphoid tissue of the head is different between rodents and humans. Rodents have concentrated lymphoid tissue at the bottom of the nasal ducts, which is either absent or disseminated in humans (119). In the human head, most of the lymphoid tissue is organized in the Waldeyer’s tonsillar ring, whereas rodents do not have tonsils (120).Non-human primates, dogs and miniature pigs provide more ideal translational models than rodents to study PD therapies. Indeed, non-human primates, dogs, minipigs, and humans all have a high prevalence of PD, and a similar PD etiopathogenesis, periodontal anatomy, oral immune system and oral microbiota (11, 117, 121). Non-human primate models are considered to bear the closest resemblance to human PD, and they have been used to study PD pathogenesis and treatment modalities including periodontal vaccines (122–124). However, research access to these animals is hindered by limited availability, high costs, ethical considerations, and difficulty in handling (117, 125). The dog offers another valuable translational PD model that is easier to obtain and maintain. Therefore it has been one of the most widely used animal models in periodontological studies, including periodontal vaccine development (113, 116, 117, 126, 127). The minipig PD model, in contrast, has not yet been used to test periodontal vaccines despite its translational value (117, 125). Nevertheless, miniature pigs have been used to assess other PD treatments such as stem-cell therapy (128), administration of pro-resolving lipid mediators (71), and photodynamic therapy (129). Therefore, dogs, non-human primates, and minipigs should be considered for future research into mucosal vaccination against PD. In these animal models, PD can be either naturally occurring or experimentally induced (e.g., with ligatures around teeth) (130). These models may facilitate the assessment of oral, intranasal, sublingual, buccal and ocular vaccination against PD (**Figure 1**). Furthermore, this may enable a better selection of antigenic targets, vaccine types, adjuvants, and delivery systems for mucosal vaccination against PD.

### Oral Vaccination

Oral vaccines have contributed substantially to the worldwide control of infectious diseases, including the near eradication of poliomyelitis (131, 132). The oral route is the most convenient and patient-accepted route of administration (133) and allows dissemination of antigen-specific lymphocytes to other mucosal tissues, such as the gastrointestinal, oral, genital, and respiratory mucosa. On the other hand, oral vaccines must overcome the gastric acid and digestive enzymes, the epithelial barrier, and the tolerogenic immune responses in order to successfully deliver antigens to the gut-associated lymphoid tissue and elicit protective immunity (134, 135).

Several studies found significant antigen-specific antibody responses in serum and saliva of mice, rats, and hamsters after peroral vaccination against antigens from human periodontal pathogens (**Table 1**). Vaccination through oral gavage or intragastric intubation generally resulted in higher levels of salivary SIgA and antibody-producing cells in mucosa-associated tissues compared to subcutaneous and intramuscular immunization (90, 95, 96). Three studies also assessed the clinical effect of these vaccinations on periodontal health, reporting protection against *P. gingivalis*-induced alveolar bone loss (103, 104) or reduced gingival swelling in a mouse gingival abscess model (105). In a first study, rats were orally vaccinated with the oral commensal *Streptococcus gordonii* that was engineered to express domains of *P. gingivalis* fimbrillin (FimA). This induced antigen-specific serum IgG, serum IgA and salivary SIgA, which protected against alveolar bone loss following *P. gingivalis* challenge (103). In another study, mice were orally immunized with the 40-kDa outer membrane protein of *P. gingivalis* (40k-OMP), adjuvanted with CpG oligodeoxynucleotides (CpG) or cholera toxin (CT). In contrast to the non-adjuvanted group, vaccination with 40k-OMP plus CpG or CT resulted in strong serum IgG and IgA, while only 40k-OMP plus CpG induced strong salivary SIgA responses. More importantly, while both groups had less alveolar bone loss following *P. gingivalis* challenge compared to the control and non-adjuvanted group, 40k-OMP plus CpG ODN provided significantly more protection than 40k-OMP plus CT (104).. The most recent of these three studies assessed the clinical effect of vaccination in a mouse gingival abscess model, where abscessation was induced by injecting a bacterial suspension in the gingiva. Vaccination was performed by oral administration of recombinant *Lactobacillus acidophilus* expressing the major outer membrane porin protein of the pathobiont *F. nucleatum* (FomA). Immunization with this live carrier induced antigen-specific serum IgG and salivary SIgA. Moreover, when the resulting serum was incubated with *F. nucleatum in vitro*, the coaggregation with *P. gingivalis* was significantly reduced. When abscesses were induced in these mice using *F.* nucleatum by itself or with *P. gingivalis*, the vaccinated mice had significantly less abscessation (105).

**Table 1:** Preclinical development of oral vaccines against periodontal disease, organized per target pathogen.

Antigen	Delivery/Adjuvant	Model	Results	Ref.
***Aggregatibacter actinomycetemcomitans***				
Fimbrial oligopeptide	Liposome IL-4 expression plasmid CT	Mouse	- Serum IgG and salivary IgA responses - Serum IgG: IM > PO > IN ^a,b^ - Salivary IgA: IN > PO >IM ^a,b^	(90)
***Fusobacterium nucleatum***				
FomA protein	Live carrier: *Lactobacillus acidophilus*	Mouse	- Serum IgG and salivary IgA responses - Abs reduce *P. gingivalis - F. nucleatum* coaggregation *in vitro* - Reduced *P. gingivalis/F. nucleatum*-induced gingival swelling	(105)
***Porphyromonas gingivalis***				
Whole cells (formalin-inactivated)		Hamster	- Serum and salivary Ab response - Serum Ab: SC > PO ^a,b^ - No significant reduction in *P. gingivalis* colonization	(96)
Fimbriae	CT	Mouse	- Serum IgM, IgG, and IgA responses - IgG and IgA responses in saliva and fecal extract - Salivary IgA and IgG: IN > PO ^a^ - Higher antibody levels with CT	(136, 137)
Fimbriae	Liposome GM-53 or MDP-Lys(L18)	Mouse	- Serum IgM, IgG, and IgA responses - Salivary IgA response - Serum IgG: SC > PO ^a,b^ - Salivary IgA: PO ≥ SC ^a,b^ - Adjuvant: GM-53 > MDP-Lys(L18)	(92, 94, 95)
FimA (residues 55-145 or 226-337)	Live carrier: *Streptococcus gordonii*	Rat	- Serum IgG and IgA responses - Salivary IgA response - Reduced *P. gingivalis*-induced alveolar bone loss	(103)
FimA (residues 1-200 or 201-337)	CTB (fusion)	Mouse	- Serum IgG and fecal IgA responses - No serum IgG and lower level of fecal IgA in absence of CTB	(138)
Hemagglutinin A	Live carrier: *Salmonella Typhimurium*	Mouse	- Antigen-specific serum antibody response	(139)
Hemagglutinin B	Live carrier: *Salmonella Typhimurium*	Mouse	- Serum IgG and IgA responses - Salivary, biliary, vaginal, and fecal IgA responses - Serum IgG: surface-expressed Ag > cytoplasm expressed Ag	(140–142)
40k-OMP	CT	Mouse	- Serum IgM, IgG, and IgA responses - Salivary, nasal, and fecal IgA responses - Serum IgG: IN > SL > PO ^a^ - Salivary IgA: IN ≈ SL > PO ^a^	(143)
40k-OMP	CT or CpG ODN	Mouse	- Serum IgG and IgA responses - Salivary IgA response - Adjuvant: CpG ODN > CT > None - Reduced *P. gingivalis*-induced alveolar bone loss	(104)

a different dosing was used per administration route.

b different adjuvants were used for different administration routes.

40k-OMP, 40-kDa outer membrane protein; Ab(s), antibody(-ies); Ag, antigen; CpG ODN, CpG oligodeoxynucleotides; CT, cholera toxin; CTB, cholera toxin subunit B; FimA, fimbrillin; FomA, Fusobacterial outer membrane protein A; GM-53 & MDP-Lys(L18), acyl derivatives of muramylpeptides; IgA/G/M, immunoglobulin A/G/M; IL-4, interleukin 4; IM, intramuscular; IN, intranasal; PO, per os;SC, subcutaneous; SL, sublingual.

Overall, the oral vaccination studies have been encouraging, but research into mucosal periodontal vaccines has recently shifted towards nasal and sublingual immunization, which generally induce higher levels of salivary SIgA (90, 136, 143).

### Intranasal Vaccination

The intranasal route is the second major mucosal route, with the nasal influenza vaccine as pioneering application in humans (131). Compared to the oral vaccination route, intranasal administration has the advantages of avoiding the gastric degradation of oral vaccines. On the other hand there exists a risk of retrograde neuronal migration of vaccine components in the olfactory or facial nerve, which can cause neural issues like transient facial nerve paralysis (144, 145). Fortunately, this risk may be mitigated by avoiding the use of certain adjuvants and antigens that are prone to neuronal accumulation, including cholera toxin and *Escherichia coli* heat labile toxin. Indeed, Du et al. reported less neuronal accumulation following intranasal immunization against PD when using antigen-fused *E. coli* maltose-binding protein as an alternative adjuvant to cholera toxin, while providing similar protection against alveolar bone loss (111).

The reviewed intranasal PD vaccination studies reported varying levels of antigen-specific antibody responses in serum and saliva of mice, rats, and dogs (**Table 2**). Eight of these studies also demonstrated various protective effects of the induced antibodies *in vitro*, including inhibited bacterial co-aggregation, decreased biofilm formation, reduced bacterial invasion of epithelial cells, and decreased pathogen viability (93, 99, 108–110, 114, 147, 148). Moreover, several *in vivo* experiments indicated that intranasal vaccination against certain antigens of *P. gingivalis, F. nucleatum*, or *Eikenella corrodens* can protect against experimentally induced alveolar bone loss or gingival swelling/abscessation in mice, rats, or dogs (91, 100–102, 108, 110–115). Nonetheless, the significant differences between nasal lymphoid tissue in rodents and humans must be taken into account when extrapolating these results (154). Furthermore, intranasal vaccination in mice is often associated with inhalation and ingestion of vaccine antigens, making discrimination between intranasal, oral, and intrapulmonary vaccination difficult (155). These limitations further highlight the unexploited value of appropriate and clinically relevant animal models.

**Table 2:** Preclinical development of intranasal vaccines against periodontal disease, organized per target pathogen.

Antigen	Delivery/Adjuvant	Model	Results	Ref.
***Aggregatibacter actinomycetemcomitans***				
Fimbrial oligopeptide	Liposome IL-4 expression plasmid CT	Mouse	- Serum IgG and salivary IgA responses - Serum IgG: IM > PO > IN ^a,b^ - Salivary IgA: IN > PO >IM ^a,b^	(90)
Serotype b-specific polysaccharide	BSA (fusion) CTB	Mouse	- Serum IgM, IgG, and IgA responses - No significant salivary Abs response - Serum IgA: IN > SC ^a,b^ - Serum IgG: SC > IN ^a,b^ - BSA (fusion) and CTB required for significant Ab induction	(97)
***Fusobacterium nucleatum***				
Whole cells (UV-inactivated)		Mouse	- Serum IgG response - Abs reduce biofilm formation and VSC production *in vitro* - Reduced *P. gingivalis/F. nucleatum*-induced gingival swelling	(109)
FomA	CT	Mouse	- Serum IgG and IgA responses - Salivary and nasal IgA responses - CT required to induce significant Ab levels	(146)
FomA	Inactivated carrier: *Escherichia coli*	Mouse	- Serum IgG response - Abs reduce *F. nucleatum* co-aggregation with *P. gingivalis*, biofilm formation and VSC production - Reduced *P. gingivalis/F. nucleatum*-induced gingival swelling	(110)
***Fusobacterium nucleatum and Porphyromonas gingivalis***				
Truncated FomA RgpA (Hgp44 domain)	FlaB (fusion)	Mouse	- Serum IgG and salivary IgA responses - FlaB (fusion) is a potent mucosal adjuvant - Divalent vaccine Abs reduced *F. nucleatum*-mediated biofilm formation, co-aggregation of *P. gingivalis* and *Treponema denticola*, and *P. gingivalis*-host cell interactions *in vitro* - Reduced *P. gingivalis/F. nucleatum*-induced alveolar bone loss: Divalent vaccine > monovalent vaccines	(114)
***Porphyromonas gingivalis***				
Fimbriae	CTB	Mouse	- Serum IgG and IgA responses - Salivary, nasal, and pulmonary IgA responses - CTB enhanced Ab titers, especially salivary IgA - Reduced *P. gingivalis*-mediated alveolar bone loss	(100)
Fimbriae	CT	Mouse	- Serum IgM, IgG, and IgA responses - IgG and IgA responses in saliva and nasal wash - Salivary IgA and IgG: IN > PO	(136)
FimA (DNA)	DNA plasmid: FimA IL-15	Mouse	- Serum IgG and salivary IgA responses - Serum IgG: IN ≈ IM - Salivary IgA: IN > IM - IL-15 enhanced salivary IgA response	(89)
FimA (DNA)	DNA plasmid: FimA CTLA4	Mouse	- Serum IgG and salivary IgA responses - Reduced *P. gingivalis*-induced alveolar bone loss - CLA4 enhanced Ab responses and alveolar bone loss reduction	(112)
FimA (DNA) Hemagluttinin 2 (DNA)	DNA plasmid: FimA HA2 ± IL15 ± CpG ODN	Rat	- Salivary IgA responses - Salivary IgA: plasmid excl. IL15 ≈ plasmid incl. IL15 < plasmid excl. IL15 + CpG ODN (30 µg) - Significantly lower levels of COX-2 and RANKL in rats vaccinated with the plasmid excl. IL15 + CpG ODN (30 µg)	(102)
FimA protein	DNA plasmid: Flt3L CpG ODN	Mouse	- Serum IgG and IgA responses - Salivary IgA response - The DNA plasmid strengthened the Ab responses - IgA inhibits *P. gingivalis* binding to salivary statherin	(147)
40k-OMP	CT	Mouse	- Serum IgM, IgG, and IgA responses - Salivary, nasal, and fecal IgA responses - Serum IgG: IN > SL > PO ^a^ - Salivary IgA: IN ≈ SL > PO ^a^ - CT required for IgA responses and strengthened IgG responses - IgG reduces co-aggregation of *P. gingivalis* and *Streptococcus gordonii*	(143, 148, 149)
40k-OMP	mCTA/LTB or CT	Mouse	- Serum IgG and IgA responses - Salivary IgA response - IgG inhibited coaggregation and hemagglutinin activities of *P. gingivalis in vitro* - mCTA/LTB and CT enhanced Ab production - Less IgE when using mCTA/LTB adjuvant compared to CT - Reduced *P. gingivalis*-mediated alveolar bone loss	(108)
Outer membrane vesicles	poly (I:C)	Mouse	- Serum IgG and IgA responses - Salivary and nasal IgA responses - Salivary IgA: IN > SC ^a,b^ - Poly (I:C) enhanced Ab responses - Serum Abs decreased *P. gingivalis* viability *in vitro* - Decreased numbers of *P. gingivalis* in the oral cavity	(93, 150, 151)
Hemagglutinin A (25-kDa antigenic region)	MBP (fusion) or CT	Mouse	- Serum IgG and IgA responses - Salivary IgA response - MBP (fusion) or CT required for Ab responses - Reduced *P. gingivalis*-mediated alveolar bone loss - Accumulation in neuronal tissues: MBP (fusion) < CT	(111)
Hemagglutinin B	MPL, GPI-0100, alum, CTB, LT, or LT (E112K)	Mouse	- Serum IgG and salivary IgA response - Vaginal IgG and IgA response - Salivary IgA: IN > SC ^a,b^ - All adjuvants enhanced Ab responses, especially the LTs - Serum IgG: surface-expressed Ag > cytoplasm expressed Ag	(98, 152)
RgpA (DNA)	HVJ envelope vector	Mouse	- Serum IgG and salivary IgA responses - Salivary IgA: IN > ID (gene gun) ^a,b^ - Reduced *P. gingivalis*-mediated alveolar bone loss	(91)
RgpA (hgp44 domain)	FlaB or FlaB (fusion)	Mouse	- Serum IgG and salivary IgA responses - Adjuvant: FlaB (fusion) > FlaB > none - Serum IgG: IN > SL ^a^ - Reduced *P. gingivalis*-mediated alveolar bone loss	(115)
Kgp (HArep domain)	CTB, CTB (fusion), MPL, or LT	Mouse	- Serum IgG response - Salivary and vaginal IgA responses - Serum IgG: SC ≥ IN ^a^ - All adjuvants enhanced Ab responses, especially the CTBs - Abs reduce *P. gingivalis* invasion of epithelial cells *in vitro*	(99, 153)
GroEL	CpG ODN	Mouse	- Serum IgM, IgG, and IgA responses - Salivary and nasal IgA responses - CpG ODN required for Ab responses - Reduced *P. gingivalis*-mediated alveolar bone loss	(101)
***Eikenella corrodens***				
Lysine decarboxylase	carbigen™	Dog	- IN immunization induced a serum IgA response that remained throughout the study period - SC immunization induced a temporary serum IgG response^b^ - No significant effect on dental plaque formation - Reduced gingivitis in both IN and SC vaccinated groups	(113)

a different dosing was used per administration route.

b different adjuvants were used for different administration routes.

40k-OMP, 40-kDa outer membrane protein; Ab(s), antibody(-ies); Ag, antigen; alum, aluminum potassium sulfate; BSA, bovine serum albumin; COX-2, cyclooxygenase-2; CpG ODN, CpG oligodeoxynucleotides; CT, cholera toxin; CTB, cholera toxin subunit B; CTLA4, cytotoxic T lymphocyte-associated antigen 4; FimA, fimbrillin; FlaB, a major flagellin of Vibrio vulnificus; Flt3L, FMS-like tyrosine kinase 3 ligand; FomA, Fusobacterial outer membrane protein A; GPI-0100, a fractionated quillaja saponin derivative; GroEL, a homolog of heat shock protein 60; HVJ, hemagglutinating virus of Japan; ID, intradermal; IgA/E/G/M, immunoglobulin A/E/G/M; IL-4/15, interleukin 4/15; IM, intramuscular; IN, intranasal; LT, heat-labile enterotoxin of Escherichia coli; MBP, maltose-binding protein of E. coli; mCTA/LTB, chimere combining subunit A of mutant cholera toxin E112K with subunit B of heat-labile enterotoxin from E. coli; MPL, monophosphoryl lipid A; PO, per os; poly (I:C), polyriboinosinic polyribocytidylic acid; RANKL, Receptor activator of nuclear factor kappa-B ligand; RgpA, Arginine-specific gingipain; SC, subcutaneous; SL, sublingual; VSC, volatile sulfur compounds.

The antigenic targets of the intranasal vaccines were very similar to those included in the oral vaccines, with *P. gingivalis* gingipains as the most used addition. Gingipains are cysteine proteases that are surface-bound and secreted, comprising RgpA and RgpB with arginine-specific activity, and Kgp with lysine-specific activity (156). They function as proteinases and transpeptidases, aiding *P. gingivalis*’ adherence, growth, development, evasion of host defenses and processing of surface-associated proteins (157–160). Moreover, Kgp has the ability to cleave IgG and IgA at specific sites within the immunoglobulin (158, 161). Our review includes five studies that assessed the effects of intranasal vaccination of murine PD models against (a domain of) gingipains (91, 99, 114, 115, 153). While different protein and DNA designs were used, each construct was able to induce significant antigen-specific serum IgG and salivary SIgA responses. Moreover, the studies that assessed the clinical effects of vaccination found significantly less experimentally induced alveolar bone loss in immunized mice (91, 114, 115). Interestingly, the most recent of these studies compared vaccination against a gingipain domain (Hgp44 domain of RgpA), a membrane protein of *F. nucleatum* (truncated form of FomA), or both. All three vaccines were able to reduce alveolar bone destruction following bacterial challenge with *F. nucleatum* and *P. gingivalis*. However, based on bone volume density, the divalent vaccine provided significantly more protection than the monovalent vaccines (114). In conclusion, the aforementioned studies support the potential of gingipains as a vaccine target and the probable benefit of multivalent periodontal vaccines, although additional research is required in appropriate PD models.

The collected data suggests that intranasal vaccination against PD generally elicits higher levels of salivary IgA compared to oral and parenteral immunization in mice and dogs (89–91, 93, 97–99, 113, 136, 143). However, to achieve these responses, intranasally administered vaccines must overcome profuse mucosal secretions, mucociliary clearance, and the relative inefficient uptake of antigens by antigen-presenting cells in the nasal cavity (162). Therefore, the efficacy of intranasal vaccination is especially dependent on adjuvants, which is highlighted by several studies where the omission of adjuvant resulted in the lack of significant antibody responses (97, 101, 111, 146, 148). Furthermore, the intranasal route seems to be the only mucosal vaccination route that tested DNA-based vaccines against PD. All four of these vaccination studies targeted antigens of the keystone pathogen *P. gingivalis* and reported strong antigen-specific salivary SIgA responses after intranasal administration of the DNA-based vaccine (89, 91, 102, 112). Moreover, three of these studies assessed the effect of the vaccination on *P. gingivalis*-induced alveolar bone loss, which was reduced in all three studies (91, 102, 112). These findings stress the unexplored potential of nucleic acid-based vaccines against PD, demanding additional research into DNA and RNA-based vaccines for PD control.

### Sublingual, Buccal, and Intraocular Vaccination

To date, there are no vaccines available that use the sublingual, buccal, or intraocular administration route, except the recently commercialized sublingual vaccine Uromune^®^ for recurrent urinary tract infections (163). Nevertheless, these mucosal vaccination routes have several advantages including the avoidance of the gastric degradation that challenges oral vaccines (131). Furthermore, these administration routes do not impose the risk of retrograde neuronal migration of vaccine components which can occur after intranasal vaccination (144, 145). On the other hand, the development of such vaccines has been hindered by certain physicochemical barriers. Sublingual and buccal vaccines are challenged by the salivary flow and constant movement of tongue and masticatory muscles, while intraocular vaccines are exposed to lacrimal fluid and palpebral movement. However, the rapid progress in vaccine delivery technology offers a promising future for these underused vaccination routes (164, 165).

Studies assessing sublingual, buccal, and ocular vaccination against PD remain very limited. A few studies assessed sublingual vaccination against PD in murine models (**Table 3**), and demonstrated similar salivary SIgA levels but significantly lower serum IgG levels compared to intranasal vaccination (115, 143). Moreover, Puth et al. found a significant reduction in *P. gingivalis*-induced alveolar bone loss in mice after sublingual vaccination against this keystone PD pathogen, although an even higher level of protection was found after intranasal administration of the same vaccine (115). Thus far, no studies have assessed the buccal administration route for PD vaccination, but one study has used the intraocular route. Shimizu and colleagues assessed the antibody response in dogs after intraocular immunization with *P. gingivalis* cell lysate incorporated in pH-sensitive fusogenic polymer-modified liposomes. This intraocular vaccination induced high titers of antigen-specific serum IgG, serum IgA, and salivary SIgA. Moreover, these salivary antibodies inhibited *P. gingivalis* adherence to HeLa cells, reduced coaggregation with the synergistic oral pathogen *Actinomyces naeslundii*, and protected human oral epithelial cells against *P. gingivalis*-induced cytotoxicity (116). While these data are encouraging for the development of mucosal vaccines against PD in dogs and humans, it should be noted that data on clinical effects of mucosal vaccination against PD in non-rodent models remains limited (113). Therefore, future research using appropriate PD animal models should assess clinical parameters such as alveolar bone loss.

**Table 3:** Preclinical development of sublingual vaccines against periodontal disease organized per target pathogen.

Antigen	Delivery/Adjuvant	Model	Results	Ref.
***Porphyromonas gingivalis***				
40k-OMP	CT	Mouse	- Serum IgM, IgG, and IgA responses - Salivary, nasal, and fecal IgA responses - Serum IgG: IN > SL > PO ^a^ - Salivary IgA: IN ≈ SL > PO ^a^ - CT required for IgA responses and strengthened IgG response - IgG reduces co-aggregation of *P. gingivalis* and *Streptococcus gordonii*	(143)
40k-OMP	Flt3L expression plasmid or CT	Mouse	- Serum IgG and IgA responses - Salivary IgA response - Flt3L expression plasmid or CT required for IgA responses - Serum Ab responses: CT > Flt3L expression plasmid - Reduced *P. gingivalis*-mediated alveolar bone loss	(106)
Hemagglutinin A (25-kDa antigenic region)	MBP (fusion)	Mouse	- Serum IgG and IgA responses - Salivary IgA response - MBP required for IgA responses - Serum Abs decreased *P. gingivalis* viability *in vitro* - Reduced *P. gingivalis*-mediated alveolar bone loss	(107)
RgpA (hgp44 domain)	FlaB or FlaB (fusion)	Mouse	- Serum IgG and salivary IgA responses - Adjuvant: FlaB (fusion) > FlaB > none - Serum IgG: IN > SL ^a^ - Reduced *P. gingivalis*-mediated alveolar bone loss	(115)
GroEL	CpG ODN or CT	Mouse	- Serum IgG and salivary IgA responses - CpG ODN or CT required for Ab responses	(166)

a different dosing was used per administration route.

40k-OMP, 40-kDa outer membrane protein; Ab(s), antibody(-ies); CpG ODN, CpG oligodeoxynucleotides; CT, cholera toxin; FlaB, a major flagellin of Vibrio vulnificus; Flt3L, FMS-like tyrosine kinase 3 ligand; GroEL, a homolog of heat shock protein 60; IgA/G/M, immunoglobulin A/G/M; IN, intranasal; MBP, maltose-binding protein of E. coli; PO, per os; RgpA, Arginine-specific gingipain; SL, sublingual.

## Opportunities

### Mucosal Vaccination Routes

Mucosal vaccination is steadily gaining interest due to their demonstrable advantages over systemic vaccination, and their increasingly efficient vaccine formulations and delivery systems (167). These advancements may accelerate the development of periodontal vaccines, which could benefit from a mucosal vaccination approach that induces both mucosal and systemic immunity in the oral cavity (88). Over the past decade, the sublingual and buccal vaccination have gained significant interest, as demonstrated by the numerous pre-clinical and clinical trials (163, 164, 168). This may inspire intensified research into sublingual and buccal vaccination against PD, which has so far received little attention compared to the intranasal and oral routes.

### Vaccine Targets

The earliest periodontal vaccines included inactivated bacteria that were easy to culture after isolation from oral sites with PD (86). Later, with the emergence of culture-independent methods, the reliance on culturability decreased and several PD-associated bacterial complexes were identified. The “red” complex comprised three species that were strongly associated with each other and with PD sites: *Porphyromonas gingivalis*, *Tannerella forsythia*, and *Treponema denticola* (169). However, while the identification was no longer culture-based, subsequent vaccine studies remained mostly limited to the easiest of these three bacteria to grow and genetically manipulate, namely *P. gingivalis* (170). Moreover, this selection approach continued to be based on the bacteria’s presence at PD sites rather than their role in the development of the disease. Nevertheless, follow-up research supported the importance of *P. gingivalis*, which is currently considered as the primary keystone pathogen in human PD (24). Similarly, a catalase-positive form of *P. gingivalis*, called *P. gulae*, is associated with PD in dogs (171).

Most periodontal vaccine studies have targeted antigens of *P. gingivalis*, while a smaller portion have focused on *Fusobacterium nucleatum* and *Aggregatibacter actinomycetemcomitans*. *F. nucleatum is* considered as a pathobiont although some strains could act as homeostatic commensals, while *A. actinomycetemcomitans* is viewed as a pathobiont that can act as a keystone pathogen in localized aggressive periodontitis (172–175). Furthermore, several other potential PD-inducing bacteria including *Filifactor alocis* and *Desulfobulbus oralis* have only recently been identified by culture-independent methods (24). Overall, additional research is needed to gain insights into the roles of the different PD-associated bacterial species in the pathogenesis. This will facilitate the selection of bacterial targets, which may become more specific as we gain better insights into the virulence factors of PD-associated pathogens. Moreover, there are increasingly efficient antigen-prediction tools that can further contribute to vaccine specificity which reduces cross-reactions, and thereby improves safety and efficacy of periodontal vaccines (176). Furthermore, these antigenic targets could be combined in multivalent vaccines, potentially further enhancing the efficacy of periodontal subunit vaccines which have so far been mostly monovalent (23).

### Vaccine Formulation and Delivery Systems

Four DNA-based vaccination studies suggest that nucleic acid-based vaccines may induce protective immunity against PD in the oral cavity (89, 91, 102, 112). Nucleic acid vaccines allow rapid, scalable, and generic production of vaccines that are efficacious at low dosage. Initial concerns about integration of exogenous DNA into the genome have subsided following clinical trials demonstrating the safety of DNA vaccines. The integration of RNA-based vaccines into the host genome is even less likely, since this would only be possible in the presence of retroviral enzymes such as reverse transcriptase and integrase (177). While both types appear safe, DNA has historically received more attention due to its higher inherent stability and lower innate immunogenicity, as well as the excellent results in rodents. However, translation to larger mammalians has been less successful. Messenger RNA (mRNA) vaccines are steadily gaining interest due to three major developments. Firstly, the use of modified nucleosides has greatly improved mRNA stability, while decreasing its innate immunogenicity (177). Secondly, there has been substantial progress in the mRNA vaccine delivery systems such as lipid nanoparticles, which has further improved stability and effectiveness of mRNA vaccines (178). The third major development occurred during the COVID-19 pandemic, when the first mRNA vaccines proved safe and highly effective against the SARS-CoV-2 virus (179). These breakthroughs may encourage the development of new nucleic acid vaccines against PD, which should no longer be limited to DNA-based vaccines.

Concurrent progress in mucosal adjuvant technologies offers additional opportunities for periodontal vaccine development. Most of the reviewed studies used bacterial adjuvants, while only a few evaluated innovative nucleic acid adjuvants (93, 101, 102, 104, 147, 150, 151, 166) and cytokine adjuvants (89, 90, 112). These adjuvants should be further assessed, but future studies should also consider particulate adjuvants such as chitosan, virus-like particles, and immune stimulating complexes, and particulate adjuvants such as chitosan. These particulate adjuvants can simultaneously act as adjuvant and mucosal delivery system (180) and might contribute to the development of mucosal periodontal vaccines. Indeed, mucosal vaccine delivery systems represents a third unexploited source that could increase periodontal vaccine efficacy. Liposomes (90, 92, 94, 95, 116), bacterial outer membrane vesicles (93, 150, 151), bacterial carriers (103, 105, 110, 139–142), and a viral carrier for DNA vaccination (91) have been successfully tested for mucosal delivery of periodontal vaccines in rodents. However, only liposomes (116) and polymer-based (113) delivery have been tested in dogs. Moreover, comparative data on the delivery systems are lacking and many other promising delivery systems, including lipid nanoparticles, have not yet been tested for mucosal vaccination against PD (167). Future research into PD vaccine delivery technology should also assess the expanding range of physical devices that localize vaccine release and/or mechanically disrupt mucosa for highly efficient delivery. Examples of highly suitable devices for mucosal PD vaccination in the oral cavity are microneedle arrays and mucoadhesive patches (181).

## Discussion

The reviewed data support the rationale behind mucosal PD vaccination as an adjunct to mechanical debridement for long-term PD control. Moreover, mucosal vaccination seems to be superior to systemic vaccination for the induction of protective immunity in the oral cavity. The reviewed preclinical studies used inactivated whole-cell vaccines, subunit vaccines, and DNA vaccines to induce immunity against PD-associated pathogens. Most vaccines were administered intranasally or orally, but a few recent studies assessed the sublingual and intraocular route. Most PD vaccines targeted *Porphyromonas gingivalis*, while a few targeted *Aggregatibacter actinomycetemcomitans*, *Fusobacterium nucleatum*, or *Eikenella corrodens*. All studies found significant increases in antigen-specific antibodies, and those assessing clinical effects also observed reduced pathological manifestations (91, 100–115). While these data are encouraging, it should be noted that all but two studies (113, 116) used rodents, which have limited translational values.

Periodontal vaccines would complement mechanical debridement by promoting pathogen-specific bacterial clearance, blocking certain virulence factors, and shifting the immune response from destructive hyperinflammation to controlled homeostatic immunity. *P. gingivalis* seems to be a very promising candidate for periodontal vaccines, due to its disproportionately large influence on the microbial community and its role in the subversion and dysregulation of the host immune response (24). This keystone pathogen can impair the host immunity through manipulation of complement and Toll-like receptor function, subversion of neutrophils and macrophages, degradation of immunoglobulins and antimicrobial peptides, interactions with dendritic cells, and “local chemokine paralysis” in epithelial cells (24, 158, 161). Successful vaccination against the involved virulence factors may prevent the dysregulation of the host’s immune response and may contribute to oral eubiosis. Although *P. gingivalis* is only one of many bacteria implicated in periodontitis, specific immunity to this keystone pathogen has been linked to protection against clinical disease in animal models such as mice, rats and non-human primates (91, 100–104, 106–108, 111, 112, 114, 115, 124, 182).

This review discusses several immunization strategies, presenting *P. gingivalis* gingipains as a promising vaccine target (114, 115, 182). Gingipains (RgpA, RgpB, and Kgp) are proteases which play an important role in the colonization, interbacterial interactions, and immune subversion by *P. gingivalis* (24, 147, 150). This is supported by the reduced alveolar bone destruction in mice and non-human primates with experimental periodontitis following vaccination that targets gingipains (91, 114, 115, 124, 182). Another study assessed the effect of anti-gingipain egg yolk antibodies as an adjunct to non-surgical periodontal therapy in humans and found improved clinical outcomes when antibody-containing gel was administered into the periodontal pockets following scaling and root planing (183). Interestingly, gingipains are also produced by *Porphyromonas gulae*, a keystone pathogen in canine PD, and treatment with a Kgp inhibitor was reported to reduce gingival swelling and periodontal pockets in dogs with naturally occurring periodontitis (184). Overall, the aforementioned studies indicate that gingipains are promising vaccine targets, although more research is needed in appropriate PD models.

Our knowledge concerning the destructive and protective immune responses in PD is still incomplete. However, T-helper17 cells seem to be involved in periodontitis pathogenesis while T-helper 2 cells are associated with protective immune responses (185–187) Moreover, salivary SIgA seems to have an important role in the maintenance of oral symbiosis and homeostatic immunity (188). SIgA antibodies are produced as the major isotype on mucosal surfaces which limits the access of microorganisms and mucosal antigens to the mucosal barrier. Furthermore, SIgA regulates the important symbiotic relationship between commensals and the host (189). The preclinical data on periodontal vaccines supports the protective role of vaccine-induced salivary SIgA, although additional research is needed (91, 100, 103–108, 110–112, 115). Considering that mucosal vaccination generally induces a stronger SIgA response than systemic immunization, the former may be more suited for vaccination against PD (88).

The potential for preventing and treating PD with mucosal periodontal vaccines is apparent, especially considering the recent progress in vaccinology which provides various opportunities. However, the available data is insufficient and difficult to interpret due to the use of rodent models. This demands further research into vaccine targets, formulations, and delivery systems *via* different mucosal vaccination routes. Moreover, the polymicrobial nature of PD calls for additional development and assessment of multivalent vaccines that can simultaneously induce antibodies against multiple pathogenic factors. These future studies should also reduce their reliance on rodent models, instead opting for more appropriate and clinically relevant animal models such as dogs, non-human primates or miniature pigs (117, 121).

In conclusion, there may be a sufficient rationale for mucosal vaccination against PD. However, the immunopathogenic complexity and polymicrobial aspect of PD appear to complicate the development of vaccines. Successful periodontal vaccines might require mucosal administration and a multivalent approach, which should be assessed in follow-up studies using appropriate animal models. Nevertheless, mucosal vaccination against PD appears feasible based on the available preclinical data.

## Author Contributions

VV and BD conceived the review article. VV wrote the manuscript in consultation with BA, BD, EC, and NS. All authors contributed to the article and approved the submitted version.

## Funding

VV is supported by a PhD scholarship from Ghent University.

## Conflict of Interest

The authors declare that the research was conducted in the absence of any commercial or financial relationships that could be construed as a potential conflict of interest.

## Publisher’s Note

All claims expressed in this article are solely those of the authors and do not necessarily represent those of their affiliated organizations, or those of the publisher, the editors and the reviewers. Any product that may be evaluated in this article, or claim that may be made by its manufacturer, is not guaranteed or endorsed by the publisher.
